# Comparing predictive abilities of longitudinal child growth models

**DOI:** 10.1002/sim.7693

**Published:** 2018-08-09

**Authors:** Craig Anderson, Ryan Hafen, Oleg Sofrygin, Louise Ryan, members of the HBGDki Community

**Affiliations:** ^1^ School of Mathematical and Physical Sciences University of Technology Sydney; ^2^ ARC Centre of Excellence for Mathematical and Statistical Frontiers (ACEMS); ^3^ Department of Statistics Purdue University; ^4^ Division of Biostatistics University of California Berkeley CA USA; ^5^ School of Mathematics & Statistics University of Glasgow, University Place Glasgow G12 8QQ United Kingdom

**Keywords:** child development, growth curve, trajectory

## Abstract

The Bill and Melinda Gates Foundation's Healthy Birth, Growth and Development knowledge integration project aims to improve the overall health and well‐being of children across the world. The project aims to integrate information from multiple child growth studies to allow health professionals and policy makers to make informed decisions about interventions in lower and middle income countries. To achieve this goal, we must first understand the conditions that impact on the growth and development of children, and this requires sensible models for characterising different growth patterns. The contribution of this paper is to provide a quantitative comparison of the predictive abilities of various statistical growth modelling techniques based on a novel leave‐one‐out validation approach. The majority of existing studies have used raw growth data for modelling, but we show that fitting models to standardised data provide more accurate estimation and prediction. Our work is illustrated with an example from a study into child development in a middle income country in South America.

## 1 INTRODUCTION

The Healthy Birth, Growth and Development knowledge integration (HBGDki) project is a collaboration funded by the Bill and Melinda Gates Foundation to integrate information from a wide range of different studies of child growth and development from across the world. While the project includes some studies from countries such as the United States and the Netherlands, the majority are from low or middle income countries. The ultimate goal of this project is to create a knowledge platform that can inform decisions about interventions in these lower and middle income countries in order to enhance child growth and improve overall health and well‐being.

Central to the HBGDki goal is understanding the factors and conditions that impact on the physical growth and development of children. Consequently, it is critical that we have reliable methods that allow the characterisation of different growth patterns. For example, we need to identify and distinguish between children who are growing successfully and those whose growth is faltering.^1^ In the cases where children do falter, we wish to quantify the timing and the extent of their recovery.^2^, ^3^, ^4^ Once we have identified methodology for characterising growth patterns, we can begin to explore the factors that predict faltering and recovery, and to explore the relationship between faltering or recovery and other outcomes such as cognitive development. 5, 6, 7

The study of human growth has long been of interest to scientists and health professionals, and growth was first linked to socio‐economic status as long ago as the early 19th century.^8^ A historical overview of growth modelling is outlined in Hermanussen and Bogin,^9^ but we are particularly interested in the modern statistically‐oriented approaches to growth trajectory modelling. One of the first such methods proposed was the LMS method,^10^ which can be seen as a precursor to the SITAR method that is in common use today.^11^ The increasing accessibility of statistical software has led to a recent surge in the use statistical growth modelling approaches to model child development^12^, ^13^ and investigate factors affecting growth.^14^, ^15^ However, the bulk of the existing literature has focused on models based on raw growth measurements, rather than modelling growth relative to some global or local standard. Most people will be familiar with the use of standardised growth charts to assess how an individual child compares with the population distribution for children of the same age and gender. These standardised charts allow us to quantify a child's relative height or weight at a particular age in the shape of a *Z* score. Different reference charts are used for male and female children so that a child's *Z* score at a given age represents their size relative to the reference population of children of the same gender and age. If we continue to monitor a child's progress as they get older, then we will obtain multiple *Z* scores at different ages, and these can be used to identify whether a child's relative growth is improving or declining over time. Standard deviation scores^16^ can be computed to quantify the change in a child's *Z* score over time, but assessing the significance of such changes is non‐trivial and requires sophisticated consideration of the expected variation in centile crossing as well as the potential impact of regression to the mean.^17^, ^18^, ^19^

In addition, standard deviation scores–based measurements are limited to evaluating the growth changes between 2 timepoints for an individual child and are unable to characterise more complex growth patterns. Many epidemiological studies, including those in the HBGDki database, involve children who have been observed at multiple timepoints, and these timepoints are not necessarily the same for each child. Therefore, in this paper, we suggest that faltering and recovery can be more extensively investigated by estimating each child's growth trajectory using longitudinal modelling techniques. The idea of extracting indicators and measures of child growth rates from fitted longitudinal models is not new. Grajeda et al^20^ model growth using linear mixed effects modelling based on regression splines. They consider several different models and derive the associated derivatives of each model to characterise child‐specific growth rates. The application discussed in their paper is based on modelling of raw growth data, and although the authors point out that the methods would also be applicable to *Z* scores, they do not test such models. One important contribution of our paper will be to explore *Z* score modelling in more detail and to provide a quantitative comparison with raw data modelling.

Section 2 provides a detailed description of the data available in the HBGDki database, including a discussion of the types of outcome data, which will be modelled. In Section 3, we provide an overview of the growth modelling literature and discuss their advantages and disadvantages in our context. In Section 4, a selection of these methods are applied to a variety of datasets from the HBDGki database, and we use a novel validation approach to test their efficacy. Section 5 outlines some additional considerations relating to growth modelling, and then we conclude with a discussion in Section 6.

## 2 DATA

The HBDGki project is an ambitious and ongoing initiative, which so far has amassed data from well over 100 studies. At the time of analysis, 21 of these studies contained data with sufficient longitudinal measures of the 2 main child growth outcomes, height (or length), and weight. In total, these longitudinal datasets contain around 800 000 observations made on over 100 000. Data from the following studies were used in this paper: Zn Trial in Burkina Faso (*bfzn*,^21^); Longitudinal Growth Study in Bangladesh (*bngd*,^22^); CMC Vellore Birth Cohort 2002 (*cmc*,^23^); Child Malnutrition and Infection Network (*cmin*,^24^); Evaluation and Control of Neglected Mucosal Enteric Infections in Childhood (*cntt*,^25^); The Consortium Of Health‐Orientated Research in Transitioning societies (*cort*,^26^); Study of Biomarkers for Environmental Enteropathy (*ee*,^27^); The Fels Longitudinal Study (*fels*,^28^); Longitudinal study of BSC in Guatemala (*gbsc*,^29^); The Global Enteric Multicenter Study (*gems*,^30^); LRTI, RSV and Influenza Cohort Study (*grip*,^27^); JiVitA‐3: Impact of antenatal multiple micronutrient supplementation on infant mortality (*jvt* 3, 31)); MRC Keneba (*knba*,^32^); Malnutrition and Enteric Disease Study (*mled*,^33^); Infant Growth in Peru (*phua*,^34^); Peru Persistent Diarrhea study (*ppd*,^35^); Promotion of Breast Feeding Interventional Trial (*prbt*,^36^); Peru Zn Fortification (*pzn*,^37^); Respiratory Pathogens Birth Cohort (*rspk*,^27^); Social Medical Survey of Children attending Child health Clinics (*smcc*,^38^); Zimbabwe Vitamin A for Mothers and Babies trial (*zvit*,^39^) children.

Table 1 provides a summary of the relevant studies, which will be considered within this paper. For data confidentiality reasons, we have labelled these datasets using letters rather than references to their location or source. The studies vary in terms of the number of growth observations per child, with some such as *cmc* having regular height or weight measurements (a median of 23 per child), while others such as *bfzn* have less frequent observation (a median of just 2 observations per child, with no child measured more than 4 times). Additionally, the studies cover a wide range of ages; for example, *gems* covers children from birth to roughly 18 months, while *cort* and *fels* measure subjects all the way to adulthood. The variety of the data makes it difficult to propose a single “one size fits all” modelling approach, but in this paper, we seek to make recommendations that allow for a degree of consistency in the analysis of the datasets. Such consistency is crucial for the final aims of this project, which include characterising the growth patterns across multiple studies and combining the results from these studies to identify global trends in growth.

**Table 1:** Summary of relevant studies within the Healthy Birth, Growth and Development knowledge integration project

			Obs Per Child			Child Age, d		
Dataset	No. of Children	No. of Obs	Min	Max	Median	Min	Max	Median
*bfzn*	7637	18983	1	4	2	168	927	541
*bngd*	197	2352	1	15	14	95	1903	804
*cmc*	373	12478	23	37	34	1	1111	558
*cmin*	3125	35506	1	37	9	1	1846	446
*cntt*	197	4405	10	41	21	1	702	116
*cort*	20510	158892	1	19	6	1	6954	718
*ee*	380	8436	2	26	23	1	1175	343
*fels*	1544	28823	1	77	16	1	6954	2746
*gbsc*	315	2548	1	13	10	119	493	269
*gems*	22545	43158	1	2	2	5	1908	1942
*grip*	203	1427	1	17	7	1	521	136
*jvt3*	27363	122139	1	6	5	1	960	92
*knba*	2954	41587	1	69	13	0	900	309
*mled*	2144	46499	1	25	25	1	732	336
*phua*	153	1839	1	16	13	1	679	185
*ppd*	412	2279	1	8	7	193	1282	628
*prbt*	16898	174233	1	14	11	1	3287	275
*pzn*	302	1140	2	4	4	153	457	265
*rspk*	278	3177	1	33	13	1	525	211
*smcc*	2027	15637	1	10	8	18	1095	280
*zvit*	14086	64867	1	10	5	1	1132	115

## 3 METHODS

There is a large literature on growth modelling, and it is not the purpose of this paper to provide an exhaustive review of these. However, in this section, we provide a broad overview of the different approaches that can be used for modelling growth data and provide some key references. Our focus lies in the characterisation of individual growth trajectories, and, therefore, we consider only methods of relevance for longitudinal studies and do not discuss the extensive literature on the analysis of cross‐sectional growth data. While the range of growth trajectory methods are quite varied, they have an underlying commonality in that they hypothesise that individual children vary stochastically about a population curve.^20^ In other words, it is assumed that there exists an overall mean curve for a particular population, and the differences between children can be explained as deviations from this mean curve.

Consider a study that observes the growth of *N* children over time in terms of a particular growth measurement. This measurement might reflect a physical characteristic such as height or weight, or may represent a mental characteristic such as a cognitive score. Suppose that the *i*th child has this growth measurement taken at a series of timepoints $t_{i1},t_{i2},\text{⋯}t_{in_{i}}$, and let *Y*_*ij*_ represents the growth measurement taken at time *t*_*ij*_. Note that there may be different numbers of growth measurements for each child, and that the measurements are not necessarily taken at regular intervals.

Clearly, the growth of a child will depend on both their age and their gender. There are 2 broad ways to account for this; either the age and gender can be built into the modelling process or the model can be based on age and gender standardised versions of the growth measurements. The majority of the papers on growth modelling work with raw growth data.^10^, ^11^, ^13^, ^14^, ^20^ Our contribution will be to explore statistical approaches, which can be used to model the standardised *Z* scores. Explicitly, modelling age and gender effects might be interesting from a biological perspective,^12^ but the trade‐off is that some of our degrees of freedom are used to capture the actual growth patterns rather than focusing on the trends. In this paper, we will compare modelling techniques based on raw and standardised data. Note that trajectories modelled under one approach can easily be converted to the other for illustrative purposes, so the purpose of our comparison is to see which form of data should be modelled on. Our standardised data are based on height‐ and weight‐for‐age *Z* scores (HAZ or WAZ) calculated with respect to the World Health Organisation standard population.^40^

This section will discuss the existing growth methodologies, with a particular focus on the 6 proposed growth models, which will be compared in Section 4.

### 3.1 Laird and Ware linear model

In an early paper that laid the groundwork for much of the last several decades of work on longitudinal growth curve modelling, Laird and Ware^41^ proposed the use of random effects as a means of characterising child‐specific departures from a global mean. Their approach allows each child to have a random intercept and slope via the following model

(1) $$Y_{ij}=\beta_{0}+\beta_{1}t_{ij}+\gamma_{0i}+\gamma_{1i}t_{ij}+\varepsilon_{ij}.$$

Here, *γ*_0*i*_ represents the *i*th child's deviation from the global intercept *β*_0_ and *γ*_1*i*_ is their deviation from the global slope *β*_1_. Here (*γ*_0*i*_,*γ*_1*i*_) are assumed to follow a joint normal distribution, independent of the error term *ε*, which also follows a normal distribution. This model is applicable on both the raw and standardised scale; in either case we would fit an individual straight line through our data for each child.

### 3.2 Laird and Ware quadratic model

It is clear that the random intercept and slope model is very simplistic and is unable to capture nuances such as growth faltering and catchup. However, it is straightforward to extend this formulation to capture more complex non‐linear trends. For example, one can add a quadratic time effect as follows:

(2) $$Y_{ij}=\beta_{0}+\beta_{1}t_{ij}+\beta_{2}t_{ij}^{2}+\gamma_{0i}+\gamma_{1}it_{ij}+\gamma_{2i}t_{ij}^{2}+\varepsilon_{ij},$$

where *γ*_2*i*_ is an additional random effect, representing the *i*th child's departure from the global quadratic term. Note that to avoid confusion, we will hereafter refer to this method as *lwquad* and use *lwlinear* to refer to the linear version outlined in the previous subsection. Higher degree polynomials can also be accounted for by adding further parameters to this formulation in a similar manner. However, such fully parametric approaches may struggle to capture the true growth trajectory, and we may be able to capture subtle aspects of the data more accurately using more flexible models. Spline‐based approaches provide a more flexible framework for modelling individual growth trajectories and have therefore been used extensively in this field.

### 3.3 SITAR

Cole et al^11^ proposed a method known as Superimposition by Translation and Rotation (SITAR), which involves each individual having a curve that is a shifted and transformed version of the mean growth curve. Shifting the curve up or down corresponds to mean changes, shifting it left or right corresponds to different growth times and the transformation of the curves.

The SITAR model is defined as follows:

(3) $$Y_{ij}=\omega_{i}+h\left(\frac{t_{ij}-\lambda_{i}}{\exp(-\kappa_{i})}\right),$$

where *ω*_*i*_, *λ*_*i*_, and *κ*_*i*_ are subject‐specific random effects, and *h* is a natural cubic spline curve with *h*(*t*) representing the mean curve.

A key advantage of the SITAR approach is that it describes each trajectory in terms of 3 biologically interpretable parameters. The parameter *ω*_*i*_ adjusts for child‐specific differences in height, *λ*_*i*_ accounts for different timing of growth spurts, and *κ*_*i*_ accounts for different durations of growth spurts. The actual growth curve *h*() forms an explicit part of the model. A consequence of this is that it is more natural to fit SITAR to the raw data, unlike the other methods outlined in this section, which can be applied to either type of data.

### 3.4 Brokenstick

Van Buuren^42^ proposed a piecewise linear model known as the “brokenstick” model. The author proposes modelling growth via a combination of linear segments with different slopes. This approach is essentially a linear spline model, where the knots are used to represent changepoints in the growth trajectory.

This model is based on a partition with 2 knots at the endpoints of our dataset, and an additional *M* internal knots that represent changepoints. Linear segments can then be fitted between each pair of knots, giving a global trajectory with a total of *M*+1 segments. A set of subject‐specific random effects are used to control each individual child's deviation from each segment of the global trajectory. The brokenstick model is outlined as follows:

(4) $$Y_{ij}=\sum_{m=0}^{M+1}\beta_{m}{\tilde{t}}_{im}+\sum_{m=0}^{M+1}\gamma_{im}{\tilde{t}}_{im}+\varepsilon_{ij},$$

where *β*_*m*_ is a fixed effect population coefficient and *γ*_*im*_ is a subject‐specific random effect for child *i*. Here, ${\tilde{t}}_{im}$, is obtained by applying a B‐spline transformation^43^ to *t*_*ij*_ to allow more flexibility in the modelling of time. The sum *ψ*_*im*_=*β*_*m*_+*γ*_*im*_ can be interpreted as the conditional mean for child *i* at the *m*th knot, and the set of *ψ*_*im*_ values can be connected by linear segments in order to model the trajectory of child *i*.

It is important to give consideration to both the number and location of the internal knots when fitting this model. We must choose a sufficient number of knots to capture the changes in growth pattern over time, but we must also avoid overfitting. The author gives some general advice that one should not select more knots than the average number of growth observations per child. The issue of the number of knots is explored more extensively in Section 5. The locations of the knots are also important to the overall accuracy of the growth trajectory estimates. The author recommends that the locations are selected to represent specific stages in a child's development, but it should be noted that this is in the context of fitting on the raw scale. This choice may be less crucial when fitting on the Z scale, because many developmental changes may already be accounted for by the transformation, and evenly spaced knots may provide a more straightforward representation of the growth trajectory.

### 3.5 Multilevel spline model

The brokenstick approach is based on linear splines, but higher degree polynomials can also be used to model growth trajectories. Durban et al^44^ proposed the use of cubic splines, thus allowing for more flexible global and individual growth trajectories. Additionally, they used penalisation as a means of reducing the impact of overfitting. A consequence of this is that they did not have to worry about knot choices when fitting the model. The penalised splines are represented as a mixed model, thus allowing for fast and computationally efficient fitting using existing mixed model software. This model is defined as follows:

(5) $$Y_{ij}=f(t_{ij})+g_{i}(t_{ij})+\varepsilon_{ij},$$

where *f* is a smooth function that represents the population trend and *g*_*i*_ is a smooth function that represents child *i*'s deviation from the population trajectory. Here, $f(t_{ij})=\beta_{0}+\beta_{1}t_{ij}+\sum_{k=1}^{K}u_{k}(t_{ij}-\kappa_{k})$, where *β*_0_ is the fixed population mean, *β*_1_ is the fixed population slope, and *κ*_1_,…*κ*_*K*_ is a set of knots on range of observed ages. The subject‐specific smooth function is defined in a similar way as $g(t_{ij})=a_{i1}+a_{i2}t_{ij}+\sum_{k=1}^{K}\nu_{k}(t_{ij}-\kappa_{k})$, where *a*_*i*1_ and *a*_*i*2_ are random effects controlling the linear deviation from the mean trend for child *i*, and the remaining term controls the non‐linear deviation from the mean curve. The choice of penalised splines for both *f* and *g*_*i*_ is more robust to the user's choice of the number of knots, because of its inbuilt penalty for overspecification of knots.

### 3.6 Functional principal components analysis

As was outlined for the previous model, the longitudinal growth data can be considered to be a form of functional data, and, therefore, techniques from the field of functional data analysis have been proposed. Xiao et al^45^ outlined the fast covariance estimation (FACE) approach, which was designed specifically for sparse longitudinal data of the form outlined in this paper. This approach assumes that the data take the form

(6) $$Y_{ij}=f(t_{ij})+h_{i}(t_{ij})+\varepsilon_{ij}.$$

This is similar in form to the penalised spline model (5), with the smooth function *f* representing the population curve and *h*_*i*_() representing individual departures from this population curve. The main difference between these models is the specification of the subject‐specific deviation terms. Model (5) uses a combination of random effects and smoothing splines to account for each child's departure from the mean, while the FACE approach in model (6) uses a stochastic process *h*_*i*_() to represent each individual's deviation from this mean curve. Here, *h*_*i*_() is considered to be a stochastic process with mean 0 and covariance function *C*(). The covariance function *C*() is estimated via a 2‐stage approach by first constructing a raw matrix and then applying a bivariate smoother. This covariance function is then used to specify *h*_*i*_() and thus identify the child‐specific deviation from the mean curve.

### 3.7 Software

As part of the HBDGki initiative, we have developed the *hbgd* software package that allows the user to fit Models (1) to (6) under consistent conditions. This package is available at https://github.com/HBGDki/hbgd.

## 4 COMPARING METHODS VIA CROSS‐VALIDATION

We wish to perform a comparison to identify which of our proposed modelling approaches perform best in terms of estimating the true growth trajectory and also predicting future growth trajectories. We also wish to determine whether modelling on the raw or standardised scale is more likely to yield accurate trajectories. Preliminary testing showed that the 2 Laird and Ware models were not competitive with the other approaches, and that SITAR often had difficulties converging when fitted to larger datasets. We therefore focus on comparing the other 3 models; brokenstick, penalised splines, and FACE. We will fit each of these models on both the *Z* scores and the raw data, giving a total of 6 different modelling approaches to compare. In an idealised setting, we might assess the performance of each modelling approach by collecting more data and making predictions for the newly enrolled subjects. Since this is generally not feasible, the true performance of each modelling approach could instead be approximated via sample‐splitting techniques. In particular, we rely on the principles of *K*‐fold cross‐validation,^46^, ^47^ evaluating the performance of each model based on out‐of‐sample (validation) subjects. This allows us to fairly assess the performance of each growth modelling approach. For a given study dataset, the *K*‐fold cross‐validation procedure is implemented as follows. We start by assigning each independent subject to only one of *K* nearly‐equal and *disjoint* partitions of the study data.

For each growth modelling approach, we then repeat the following procedure *k*=1,…,*K* times:

- We construct a validation set that consists only of the *k* *t* *h* partition of the subjects and combine all the remaining *K*−1 partitions into a training set.
- We fit a specific growth model based on the training set alone.
- For each child in the validation set, we remove a single growth measurement (holdoutp). For each validation subject, we then use the results from the model fitted on the training dataset to predict the holdout measurement, using the subject's remaining growth measurements (non‐holdouts) as predictors.
- We then evaluate the accuracy of these predictions for each subject in the validation set by comparing their predicted and holdout growth measurements using the mean squared error (MSE).

The predictive accuracy of each model is then computed as the average of the MSE across all *K* validation sets. This *K*‐fold cross‐validation procedure allows us to assess the performance of a given growth model based for every subject available in the data, since each subject gets a chance to be a part of only one of the *K* validation sets.

Let **y**=(*y*_1_,…,*y*_*m*_) be a vector containing the observed values of our held out data from *m* children, and let $\hat{y}=(\hat{y_{1}},\text{⋯},\hat{y_{m}})$ be the vector of predictions for those values. Then the MSE is given by

$$\text{MSE}=\frac{1}{m}\sum_{i=1}^{m}{\left(y_{i}-{ŷ}_{i}\right)}^{2}.$$

A lower MSE suggests that a model did a good job of accurately predicting the value of the removed observation. Note that to ensure consistency, the MSEs were always calculated on the Z scale. In the cases where the models were fitted on the raw data, we transformed the resulting trajectory to the Z scale in order to calculate the MSE. The Z transformation is monotonic, and, therefore, our results are not affected by our decision to calculate the MSEs on the Z scale rather than the raw scale.

We note that an alternative approach for testing the performance of a given modelling approach would be by making predictions for every available growth measurement of each validation subject. For example, one could implement a procedure that for a given validation subject would start by choosing the first available growth measurement as a holdout (ie, removing it), then making predictions using the rest of the available growth measurements, and so on, until the last available growth measurement on that subject has been used as a holdout. By averaging the subject‐specific loss across all holdout predictions, one might be able to obtain a more accurate assessment of the model performance. However, given the size and the number of the datasets considered in this work, such a procedure is currently computationally impossible to implement. Thus, we restrict ourselves to only using a single holdout observation on each validation subject. Nonetheless, our presented *K*‐fold cross‐validation procedure remains valid and provides a fair assessment of the performance of various growth modelling approaches. In particular, the above described cross‐validation procedure assesses the model generalisability for imputation of the missing growth measurements for a new (unseen) subject, given the available growth information on that subject.

Two different approaches were used for selecting the observations, which are held out for each child as part of the double validation dataset; a “random value” approach and a “last value” approach. For the random value approach, we randomly selected the held out observation from the set of all observations for the child. This approach tests the accuracy of the overall model fit, by focusing on how well it can interpolate at unobserved timepoints. A similar approach was outlined by Grajeda et al,^20^ who randomly removed 20% of observations per child. However, we also include a novel “last value” approach, which involves removing the final observation for each child, ie, the observation at which the child is oldest. This approach tests the predictive ability of the models, with a particular focus on the kind of short‐term extrapolation which such models could realistically be used for. Such future prediction is particularly important in the context of the HBGDki project, where we may frequently wish to use a child's observed trajectory to make inference about the effects of an intervention on future growth.

We note that the quality of fit and predictive ability of these methods could also have been tested using a simulation study with known parameters. However, in this case, we already having such a varied range of real datasets at our disposal, and we felt that it was preferable to use this cross‐validation approach. This also means that the conclusions we draw here are directly applicable to the datasets on which we wish to perform our analysis. The cross‐validation approach that we outline here is by no means restricted to the datasets or methods discussed in this paper; similar methods could be applied to test different models and/or different datasets within any longitudinal modelling context.

### 4.1 Illustrative example—cntt

As discussed in Section 2, we are studying 21 different datasets with longitudinal growth measures, and Section 4.2 will summarise the results obtained from each of our 21 datasets. However, for the purposes of illustration, we will present detailed results from the cntt dataset in this section. This dataset contains height and weight measurements from 197 children in a low income country. A total of 4405 height observations were taken over the study period, with a median of 21 observations per child.

**Figure 1:**
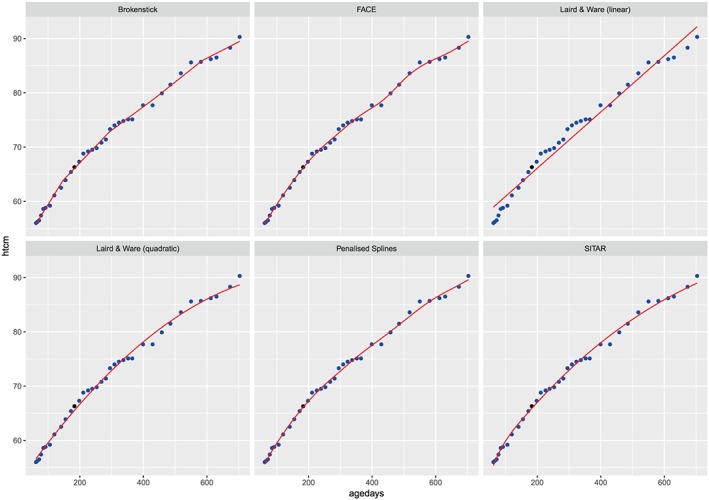
Fitted growth trajectory of a single child based on fitting each of our 6 models on the raw scale. The blue dots are the data, the black dot is the held out datapoint, and the red line is the model fit. FACE, fast covariance estimation; SITAR, Superimposition by Translation and Rotation [Colour figure can be viewed at wileyonlinelibrary.com]

**Figure 2:**
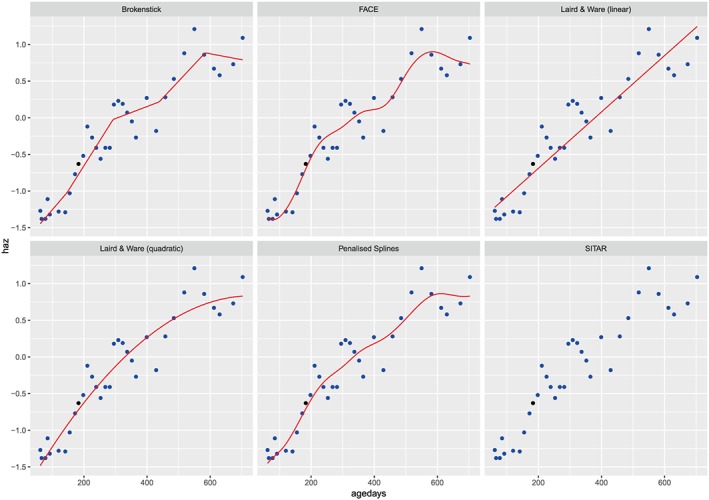
Fitted growth trajectory of a single child based on fitting 5 of our models on the Z scale (SITAR was not fitted on this scale). The blue dots are the data, the black dot is the held out datapoint, and the red line is the model fit. FACE, fast covariance estimation; HAZ, height‐for‐age Z score; SITAR, Superimposition by Translation and Rotation [Colour figure can be viewed at wileyonlinelibrary.com]

Figures 1 and 2 display the fitted growth trajectories of a single randomly selected child under each of our eleven proposed modelling strategies. In each case, we used the random holdout approach and fitted the model on the remaining data. For each panel of the plots, the points represent observed HAZ scores for the child, while the line is the fitted trajectory under the selected model. The filled point is the one that was held out for validation. Figure 1 displays the cases where the models were fitted on the raw data, while Figure 2 is based on the *Z* scores. Note that the third panel of Figure 2 does not have a fitted line because we cannot fit the SITAR model on the *Z* scores.

Figure 1 shows that the Laird and Ware approaches are not flexible enough to model a sensible growth curve based on these data. Each of the other 4 models appear to do a reasonable job of estimating the growth trajectories of the children. In Figure 2, we identify more nuanced differences between the models as a result of the transformation of the data. It appears that the brokenstick and face approaches perform best in terms of how well they predicted the holdout value. These models have more flexibility to account for this child's fluctuation in HAZ score between the ages of 400 and 600 days. The penalised spline model provides a reasonable fit but appears to be slightly too smooth to capture this fluctuation, while the Laird and Ware models are unable to capture particular feature of the child's growth trajectory and perform poorly as a result.

### 4.2 Cross‐validation

The comparison process outlined in Section 4.1 was repeated on each of the other 20 datasets. Each of these datasets have different features, and we are interested in comparing the methods in terms of MSE to test how robust each method is to different data structures.

The results of the random value validation approach are shown in Table 2, with the lowest MSE for each dataset displayed in bold. Across the 21 datasets, we can see that the brokenstick and FACE approaches generally provide lower MSE values than any of the other approaches whether they are fit on the *Z* score or raw scale. We note that for the random holdout approach, fitting on the *Z*‐score scale tends to provide lower MSE values. This is unsurprising, since fitting on the raw scale will typically use up some of our degrees of freedom on the overall curve fit, rather than just focusing on an accurate fit relative to a standard growth curve.

**Table 2:** MSE results for random value holdout cross‐validation ^a^

	Brokenstick (raw)	Brokenstick (Z)	FACE (raw)	FACE (Z)	Penalised Splines (raw)	Penalised Splines (Z)
*bfzn*	0.17	**0.16**	0.17	0.17	1.58	0.19
*bngd*	0.10	0.06	0.10	**0.06**	1.37	0.38
*cmc*	0.36	0.27	**0.23**	0.24	0.26	0.29
*cmin*	0.55	0.29	**0.27**	0.27	0.32	0.32
*cntt*	0.07	0.05	**0.04**	0.04	0.07	0.07
*cort*	3.14	**0.50**			1.05	0.61
*ee*	0.21	0.18	0.12	**0.11**	0.16	0.16
*fels*	1.18	**0.14**			0.25	0.14
*gbsc*	0.07	0.07	0.06	**0.06**	2.01	0.17
*gems*	0.36	**0.34**			0.36	0.36
*grip*	0.44	0.42	**0.40**	0.41	0.50	0.51
*jvt3*	0.89	**0.40**			0.42	0.42
*knba*	**0.59**	1.20			0.62	0.81
*mled*	0.22	**0.19**			0.19	0.19
*phua*	0.18	0.13	0.12	**0.12**	1.03	0.16
*ppd*	0.06	0.06	**0.06**	0.06	2.11	0.13
*prbt*	1.55	0.91			0.84	**0.81**
*pzn*	0.08	0.08	0.08	**0.07**	3.07	0.12
*rspk*	0.54	0.52	0.47	**0.46**	0.56	0.54
*smcc*	0.35	0.26	0.26	**0.26**	4.37	0.58
*zvit*	1.44	1.18			1.18	**1.17**

Abbreviations: FACE, fast covariance estimation; MSE, mean squared error. ^*a*^The lowest MSE for each dataset is displayed in bold.

We can see that FACE provides better estimates than brokenstick for 12 datasets, while there are 7 datasets where the brokenstick approach is more effective. In general, brokenstick appears to work better when there a low number of observations per child, which makes it more difficult for FACE to accurately estimate the necessary principal components. There were a total of 8 datasets (*cort, fels, gems, jvt3, knba, mled, prbt and zvit*), where FACE was unable to provide a successful fit because of the large size of the dataset. Fast covariance estimation is a more computationally complex approach than brokenstick and is thus more likely to run into such issues.

The relative performance of the modelling approaches was similar under the last value validation approach, as shown in Table 3. However, in this scenario, there was less of a difference in performance between the raw scale and the *Z*‐score scale. This is likely to be because we are extrapolating from our data, which is likely to lead to larger deviations from the truth. If we are fitting on the Z scale, these larger errors will be inflated when transforming to the true curve.

**Table 3:** MSE results for last value holdout cross‐validation ^a^

	Brokenstick (raw)	Brokenstick (Z)	FACE (raw)	FACE (Z)	Penalised Splines (raw)	Penalised Splines (Z)
*bfzn*	**0.16**	0.16	0.17	0.17	0.82	0.18
*bngd*	0.08	0.10	0.07	**0.07**	1.17	0.38
*cmc*	0.10	0.15	**0.07**	0.13	0.17	0.30
*cmin*	0.31	0.25	**0.25**	0.25	0.29	0.35
*cntt*	**0.04**	0.04	0.04	0.04	0.07	0.07
*cort*	0.94	**0.50**			0.59	0.56
*ee*	0.04	0.06	**0.04**	0.04	0.06	0.06
*fels*	0.41	**0.14**			0.27	0.18
*gbsc*	**0.06**	0.06	0.06	0.06	0.91	0.12
*gems*	0.34	**0.33**			0.35	0.36
*grip*	0.36	0.36	0.36	**0.36**	0.44	0.44
*jvt3*	0.66	**0.46**			0.50	0.50
*knba*	**0.50**	0.57			0.55	0.60
*mled*	**0.13**	0.14			0.14	0.14
*phua*	0.16	**0.16**	0.24	0.30	0.60	0.21
*ppd*	**0.10**	0.10	0.10	0.10	1.71	0.16
*prbt*	**0.59**	0.62			0.70	0.59
*pzn*	0.11	0.11	0.11	**0.11**	0.74	0.15
*rspk*	0.51	0.51	**0.49**	0.49	0.56	0.55
*smcc*	0.72	0.72	0.71	**0.71**	2.82	1.49
*zvit*	1.11	**1.02**			1.04	1.03

Abbreviations: FACE, fast covariance estimation; MSE, mean squared error. ^*a*^The lowest MSE for each dataset is displayed in bold.

Again, FACE provides the lowest MSE for most of the datasets but was unable to provide a fit for the very large datasets. Overall, it appears that the FACE approach represents the most accurate of the modelling approaches discussed here, both in terms of internal and external prediction. However, the brokenstick approach provides a credible alternative, and may prove to be particularly useful in cases where we have larger datasets, or where the number of observations per child is very low. The brokenstick approach is likely to work successfully on a wider range of datasets, and as a result, we recommend it as the optimal modelling approach.

**Figure 3:**
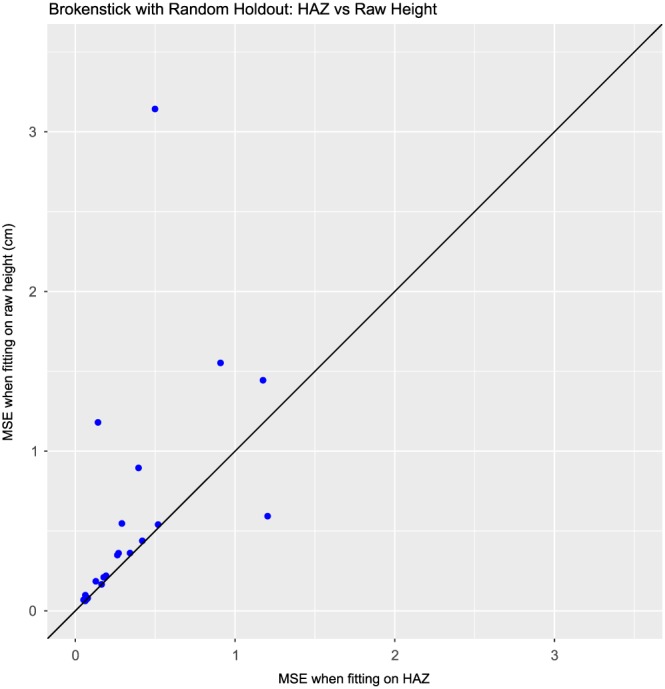
Comparison of results from fitting the brokenstick model on raw data and on Z‐transformed data. Each point represents the MSE values obtained from random holdout on 1 dataset. HAZ, height‐for‐age Z score; MSE, mean squared error [Colour figure can be viewed at wileyonlinelibrary.com]

**Figure 4:**
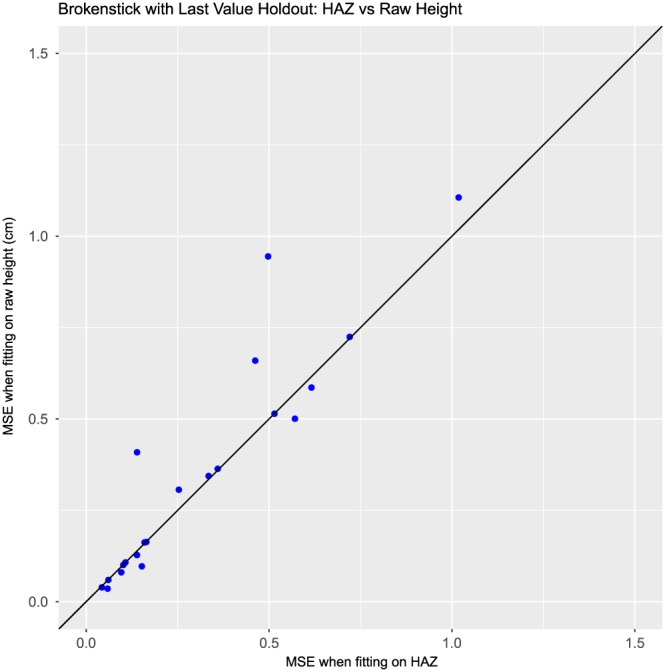
Comparison of results from fitting the brokenstick model on raw data and on Z‐transformed data. Each point represents the MSE values obtained from last value holdout on 1 dataset. HAZ, height‐for‐age Z score; MSE, mean squared error [Colour figure can be viewed at wileyonlinelibrary.com]

We are also interested in comparing the models fitted on the Z scale to those fitted on the raw data. Figure 3 presents a comparison of the MSEs obtained from modelling raw and Z‐scale data using the brokenstick model with random holdout. Each point represents a single dataset, and the x‐axis displays the MSE from fitting on the Z scale, while the y‐axis displays the MSE from fitting on the raw data. We can see that almost all of the points lie above the line of equality, which means that the MSEs were lower when we fitted the data on the Z scale. Figure 4 is a similar plot for the last value holdout. Here, we see that most of the points lie on or near the line of equality, though there are a handful of points that lie well above the line. We can see that, in general, fitting on the Z scale leads to more accurate estimation, and as a result, we recommend fitting our models on the Z scale in all cases.

## 5 FURTHER EXTENSIONS

In Section 4, we compared 3 models across 2 different fitting styles on a total of 21 datasets. This represents a very thorough and rigourous exploration of fitting techniques, but nonetheless there are other issues that must be considered in our analysis.

Each of the 3 most best performing approaches outlined in this paper rely on the user selecting a number of knots in advance. In the analysis in the previous section, we selected these parameters using the inbuilt model defaults, which deterministically select the number and range of parameters based on the size and age range of the dataset. However, it is also important to test how sensitive the results are to these choices. We therefore repeated the analysis on dataset E using various different parameter choices, and the results of these tests are outlined in Tables 4, 5, 6. We see that within each method, the MSE remains fairly stable regardless of the number of knots selected, with only a couple of exceptions. We note that the MSE is slightly higher for the brokenstick model with just 3 knots, which is likely because we do not have enough flexibility to accurately model the data. We also see that the penalised spline model performs more poorly in the cases where we have a larger number of subject‐specific splines, which is likely down to overfitting. However, we see that the results for this model are constant across different numbers of population level knots, which is expected because of the inbuilt penalisation term in the model. These results suggest that our validation test is not hugely sensitive to the number of knots selected, and that in most cases the inbuilt model defaults for brokenstick or FACE will be suitable.

**Table 4:** Mean squared error for brokenstick model with different numbers of knots

Knots	Random Holdout	Last Value Holdout
3	0.083	0.088
4	0.059	0.050
5	0.054	0.042
6	0.051	0.042
7	0.049	0.044

**Table 5:** Mean squared error for fast covariance estimation model with different numbers of knots

Knots	Random Holdout	Last Value Holdout
4	0.051	0.046
6	0.049	0.043
8	0.045	0.044
10	0.043	0.043
12	0.042	0.043

**Table 6:** Mean squared error for penalised spline model with different numbers of knots

Population Knots	Individual Knots	Random Holdout	Last Value Holdout
5	3	0.120	0.077
5	5	0.111	0.066
5	7	0.230	0.203
10	3	0.120	0.076
10	5	0.112	0.065
10	7	0.227	0.205
15	3	0.122	0.077
15	5	0.112	0.066
15	7	0.228	0.205

One of the key aims of this paper is to accurately characterise growth trajectories in order to explore the relationships between growth faltering and other outcomes. To do this, we must extract sensible indicators of growth from these trajectories, for example, mean growth over a particular time period, number of days in a particular growth state, or indicators relating to growth derivative. One measure of particular interest is the mean derivative over the first year, which acts as an indicator of the rate of growth during a formative change in a child's life. Future work will explore the relationship between this growth measure and cognitive function across multiple studies.

## 6 DISCUSSION

In this paper, we outlined a thorough comparison of a variety of commonly used methods for characterising child growth trajectories. We tested 3 different models across 21 datasets from the HBDGki database in terms of the accuracy of their model fit and also their ability to predict future growth patterns. These datasets had different characteristics in terms of the number of children and frequency of measurements, but our results showed that 2 models, brokenstick and FACE were consistently the best performing approaches. The FACE model provided slightly better estimation overall but had some difficulties fitting on larger datasets and also those with a very small number of observations per child. The brokenstick approach was more robust in these circumstances because it is a less computationally complex model.

We were also interested in whether it was more beneficial to use standardised *Z* scores or to fit on the original scale, and, therefore, we tested our models on both types of data. We identified that the *Z*‐score models were superior in terms of accurate fits, and that there was little difference between the approaches in terms of predicting future growth. One of our overall goals is to provide an integrated modelling framework for all of these datasets, and, therefore, it is important to have consistency in our modelling approaches. As a result, we recommend the use of the brokenstick model with standardised *Z*‐score data. Aside from the accuracy of the fit, another key advantage of the brokenstick model is that it is easier to fit and provides easily interpretable estimates of child growth trajectories. There will certainly be other potential modelling approaches out there, and this recommendation is based only on the methods, which we have studied. A key advantage of our modelling framework is that any potential new method could easily be incorporated and compared with the existing methods.

The work presented in this paper may motivate future work in the area of growth modelling. It is possible to use indicators extracted from our growth trajectories to investigate the effects of growth faltering on other outcomes. It is also possible to use these indicators to consider factors which may lead to growth faltering in the first place. This work could also have implications for the design of future epidemiological studies. We have identified sensible techniques for accurately modelling growth trajectories and have shown that they still perform well on sparse datasets. It may therefore be possible to design more efficient studies with a smaller number of measurements per child, while still retaining the ability to accurately model growth.

Here, we have presented a set of univariate modelling approaches, which can be used for height or weight data, but, of course, one could also consider a multivariate approach, which models for height and weight simultaneously. The biggest challenge for such an approach is the added computational complexity; we have already noted that some of the existing univariate approaches struggle with larger datasets. There may also be some scope for developing a meta‐regression approach, which would allow us to account for the heterogeneity between studies when combining our results. Here, we focused on characterising growth patterns for individual children based on population mean trends, but if we wish to make inferences about other quantities such as lower or upper quantiles then quantile regression approaches could be considered.

The goal of the HBGDki project is to integrate information from a variety of studies from across the world in order improve overall health and well‐being in children. It is therefore crucial that we identify accurate and reliable models for characterising growth trajectories in order to distinguish between children who have healthy growth and those whose growth is faltering. This allows us to explore factors that predict faltering, and also the effect of poor growth on future health, thus providing a framework for influencing decision making both in the field and at the governmental level.
